# Global analysis of sRNA target genes in *Mycoplasma hyopneumoniae*

**DOI:** 10.1186/s12864-018-5136-5

**Published:** 2018-10-23

**Authors:** Tiago Ebert Fritsch, Franciele Maboni Siqueira, Irene Silveira Schrank

**Affiliations:** 1 Centro de Biotecnologia, Programa de Pós-Graduação em Biologia Celular e Molecular, Universidade Federal do Rio Grande do Sul (UFRGS), Porto Alegre, RS Brazil; 2 Departamento de Biologia Molecular e Biotecnologia – Centro de Biotecnologia, Universidade Federal do Rio Grande do Sul (UFRGS), Av. Bento Gonçalves 9500, P. 43421, C.P. 15005, CEP, Porto Alegre, RS 91501-970 Brazil

**Keywords:** Small RNA, sRNA target gene, *Mycoplasma hyopneumoniae*, Oxidative stress; heat shock, Gene expression

## Abstract

**Background:**

Small RNAs (sRNAs) are noncoding molecules that regulate different cellular activities in several bacteria. The role of sRNAs in gene expression regulation is poorly characterized in the etiological agent of porcine enzootic pneumonia *Mycoplasma hyopneumoniae*. We performed a global analysis of the sRNAs, sRNA target genes and regulatory elements previously identified in their genome and analyzed the expression of some sRNAs and their target genes by quantitative RT-PCR (qPCR) in three different culture conditions.

**Results:**

Seven of the 145 sRNA target genes are organized as monocistronic genes (mCs) while the other 138 sRNA target genes are organized into transcriptional units (TU). The identification of transcriptional regulatory elements (promoter motif, DNA repeat sequence or intrinsic terminator) was verified in 116 of the 145 sRNA target genes. Moreover, the 29 sRNA target genes without regulatory elements revealed the presence of at least one regulatory element in the boundaries of the TU or in other internal genes of the TU. We verified that 16 sRNAs showed differential expression, seven in heat shock condition and 14 in oxidative stress condition. Analysis of the differential expression of the sRNA target genes showed that the tested sRNAs possibly regulate gene expression. The sRNA target genes were up- or down-regulated possibly in response to sRNA only under oxidative stress condition. Moreover, the sRNA target genes are involved in diverse processes of the cell, some of which could be linked to transcription processes and cell homeostasis.

**Conclusion:**

Our results indicate that bacterial sRNAs could regulate a number of targets with various outcomes, and different correlations between the levels of sRNA transcripts and their target gene mRNAs were found, which suggest that the regulation of gene expression via sRNAs may play an important role in mycoplasma.

**Electronic supplementary material:**

The online version of this article (10.1186/s12864-018-5136-5) contains supplementary material, which is available to authorized users.

## Background

Bacterial small RNAs (sRNAs) are extremely diverse and considered important components in the mechanism of gene regulation in bacteria, at the transcriptional, post-transcriptional or translational levels [1–4]. The majority of sRNAs described in genome-reduced bacteria correspond to the trans-encoded sRNAs located in intergenic regions [5]. These sRNAs may have multiple targets in the genome and could regulate the expression of a target gene, altering the mRNA stability or translation process by base-pairing [6, 7] and in some bacteria, the pairing and regulation could be mediated by several RNA-binding proteins [8].

Mycoplasmas are bacteria of the class Mollicutes, characterized by the absence of a cell wall and a reduced genome with limited biosynthetic metabolism [9]. Mycoplasmas lack the majority of known transcription factors and regulatory pathways compared with other bacteria. The transcription factors (TFs) represent approximately 2.5% of the total number of genes in mycoplasma species, representing proportionally half of those found in bacterial models like *Escherichia coli* [10]. However, mycoplasmas maintain the ability to respond to a variety of environmental and metabolic stresses [11–13], suggesting the presence of alternative transcriptional regulatory mechanisms.

*Mycoplasma hyopneumoniae* is the etiological agent of porcine enzootic pneumonia, a disease with global distribution and is considered a major cause of economic loss in the pig industry [14]. The genomes of several strains have been sequenced and analyzed. The genome of *M. hyopneumoniae* 7448 presents a unique sequence coding for sigma factor and a few transcriptional regulatory proteins [15]. *M. hyopneumoniae* genes are organized in large transcriptional units (TUs) that are continuously transcribed (co-transcribed), with each TU being transcribed in the same direction, with no intervening genes transcribed in the opposite [16, 17]. The occurrence of promoter motifs [18, 19], intrinsic terminators [20] and DNA repeat sequences [21] has already been described in this species. These regulatory elements are located both at the boundaries of TUs and also in the internal genes between several TUs. The transcription of TUs is a highly dynamic process that is mainly regulated at initiation and termination, being able to adapt in response to changing conditions, generating large transcripts in some conditions, while producing short transcripts in others [22]. The presence of regulatory elements inside the TUs suggests that these elements may have an important role in gene regulation in response to stress conditions in mycoplasmas.

In *M. hyopneumoniae* 7448, our group identified 47 different intergenic sRNAs and their target genes by in silico approaches [23]. The sRNAs were predicted in the intergenic region of the genome by a minimum free energy algorithm [23]. The expression of all tested sRNAs was validated by stem-loop reverse-transcription PCR methodology, and some of them were found to be differentially transcribed in different culture conditions [23]. The presence of other classes of noncoding RNAs has been found in Mycoplasmas, such as *cis*-encoded antisense RNAs [24] and transcription start site-associated RNAs (tssRNAs) [25]. The interaction between an intergenic sRNA and its targets has already been experimentally validated in the genome-reduced bacteria *Rickettsia conorii* [26]. However, the comprehension of the noncoding RNA regulatory mechanisms is very limited in mycoplasmas. The tssRNAs have been hypothesized to prevent transcription elongation until the correct RNAP complex has been assembled [25]. In addition, sRNAs possibly participate in the degradation of the mRNA by base-pair interaction, thereby controlling the availability of mRNA [5].

In this study, we have analyzed the regulation of the predicted sRNA target genes and their correlation with the expression of sRNAs in *M. hyopneumoniae*. Therefore, we have performed a global analysis of the sRNAs, sRNA target genes and regulatory elements previously identified in their genome. For this purpose, we have mapped the presence of promoters, DNA repeat sequences, and intrinsic terminators in genes coding for sRNAs and in the target genes predicted for each sRNA. Moreover, we analyzed the expression of some sRNAs and their target genes by quantitative PCR (qPCR) in three different culture conditions.

## Methods

### In silico analysis of transcription regulatory elements

The in silico analysis of transcription regulatory elements was performed in *M. hyopneumoniae* 7448 genome (INSDC AE017244.1). The identification data previously described for promoter motifs [19], intrinsic terminators [20], DNA repeats [21], and sRNAs [23] were utilized in this study to evaluate their role in transcription regulation. Each regulatory element was manually mapped in the respective predicted sRNA target gene using *Artemis* software [27]. This approach took into account the *M. hyopneumoniae* 7448 genomic organization, where the genes are organized into 41 monocistronic genes (mCs) and 121 transcription units (TUs) [16, 17]. In this way, the elements were positioned and compared according to their genomic position.

### Culture conditions and RNA isolation

*M. hyopneumoniae* strain 7448 was cultivated in three different culture conditions. In the standard condition, bacteria were grown in 30 ml of Friis media [28] at 37 °C for 24 h with gentle agitation in a roller drum. A heat shock stress condition was performed by incubation of the standard cultures (after the initial 24 h at 37 °C) at 30 °C for 2 h, and then shifting to 42 °C for 30 min [11, 23]. The oxidative stress condition was achieved by the addition of 1% hydrogen peroxide to the standard cultures (after the initial 24 h at 37 °C) followed by incubation at 37 °C for 15 min [12, 23]. Transcriptional response induction in stress cultures conditions was previously confirmed by Breyer et al. [29].

Cells were pelleted by centrifugation at 3360×g for 20 min and washed with DEPC-treated water. Total RNA isolation was performed with TRIzol® Reagent (Invitrogen) according to the manufacturer’s instructions, including DNase I digestion with 50 U of DNase I (Thermo Scientific). DNA absence was monitored by PCR assays. Extracted RNA was analyzed by gel electrophoresis and quantified in the Qubit™ system (Invitrogen).

### Quantitative PCR experiments

The reverse-transcription (RT) reactions were performed with 700 ng of total RNA, 132.5 ng of pd.(N)_6_ random hexamer (GE Healthcare) and 10 mM of deoxynucleotide triphosphates. The mixture was heated to 65 °C for 5 min and then incubated on ice for 5 min. M-MLV-RT 5× reaction buffer (Invitrogen) and 0.1 M dithiothreitol (DTT) was added to the reaction and incubated at 37 °C for 2 min. Then, 200 U M-MLV reverse transcriptase (M-MLV RT) was added and the reaction was incubated at 25 °C for 10 min, followed by 50 min at 37 °C and finally for 15 min at 70 °C for enzyme inactivation. A negative control was prepared in parallel, differing only by the absence of the M-MLV RT enzyme.

A quantitative PCR (qPCR) assay was performed using 1:7 cDNA as template and Platinum SYBR Green qPCR SuperMix-UDG (Invitrogen) on 7500 Fast Real-Time PCR System (Applied Biosystems). The qPCR reactions were carried out at 90 °C for 2 min and 95 °C for 10 min followed by 40 cycles of 95 °C for 15 s and 60 °C for 1 min each. The primers were designed to amplify sRNAs and sRNA target genes using *Vector NTI Advance 10* (Invitrogen). The primer sequences are described in Additional file 1. The specificity of the synthesized products and the absence of primer dimers were visualized using a melting curve analysis for each reaction. The amplification efficiency for each primer pair was calculated using the LinRegPCR software application [30] and the mean efficiency values for each primer were added to Additional file 1. This efficiency value was used for the quantification analysis.

The relative expression of sRNAs and sRNA target genes was evaluated in the three different culture conditions. The relative expression was calculated by the 2^-ΔΔCt^ method [31]. The threshold cycle (CT) values were normalized to the reference gene MHP7448_0333 [19, 20]. Three technical and biological replicates were undertaken for each sRNA or gene evaluated. Statistical analyses were performed using GraphPad Prism 6 software by One-way ANOVA followed by Tukey’s multiple comparison test. The conditions to statistical test were fulfilled and differences were considered statistically significant at *P* < 0.05.

## Results

### sRNA target gene organization

Analysis of the *M. hyopneumoniae* 7448 genome showed the presence of 47 sRNAs and 145 putative sRNA target genes (Additional file 2) [23]. To investigate the possible role of sRNAs in gene regulation, a global analysis was performed based on the available data of the sRNA target genes and transcriptional regulatory elements using both in silico and experimental approaches.

The *M. hyopneumoniae* genes are organized into 41 mCs and 121 TUs containing two or more genes [16, 17]. The location of the 145 sRNA target genes was analyzed according to the gene organization in the genome. Seven of the 145 sRNA target genes are organized as mCs, representing 17% of the total number of mCs in the genome. The other 138 sRNA target genes are organized into TUs, being distributed into 77 different TUs representing 64% of the total number in the *M. hyopneumoniae* genome. In general, TUs contain only one sRNA target gene, although some TUs have up to 6 sRNA target genes. This is the case for TU_84, where all four genes that composed it, MHP_0486, *mgtE*, MHP_0488 and MHP_0489, are targets of different sRNAs (Additional file 2). Considering the location of the sRNA target genes in the TUs, our results demonstrated that 26 sRNA target genes are the first gene in the TU, 24 sRNA target genes are the last gene in the TU and the other 88 sRNA target genes are internal genes within the TUs (Additional file 3).

Usually, the regulation of transcription occurs mainly at the boundaries of the transcription units; this mechanism could be present in the mycoplasma genome as the genes are co-transcribed [5, 16]. However, alternative transcripts may also be produced by the presence of internal transcription regulators within the transcription units. Moreover, the most common mechanism of gene transcription is related to the presence of typical DNA sequences of promoters, repetitive elements and intrinsic terminators. Therefore, the search for transcription regulatory elements was performed both at the sRNA single gene targets and transcription units containing sRNA target genes.

**Fig. 1:**
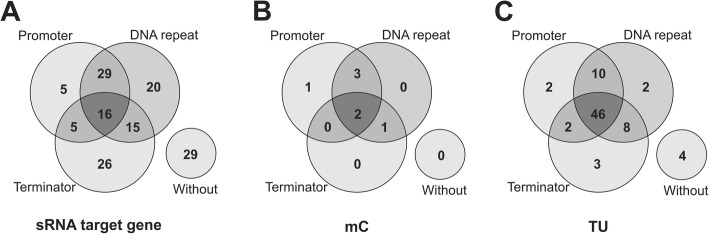
Number of genes or transcriptional units with transcription regulatory elements. **a** Number of genes containing promoter motifs, DNA repeat sequences and intrinsic terminators considering the 145 sRNA target genes. **b** and **c** Presence of transcription regulatory elements considering the organizational structure of transcriptional units with sRNA target genes, B represents the 7 mC sRNA target genes and C represents the 77 TUs with sRNA target genes

**Fig. 2:**
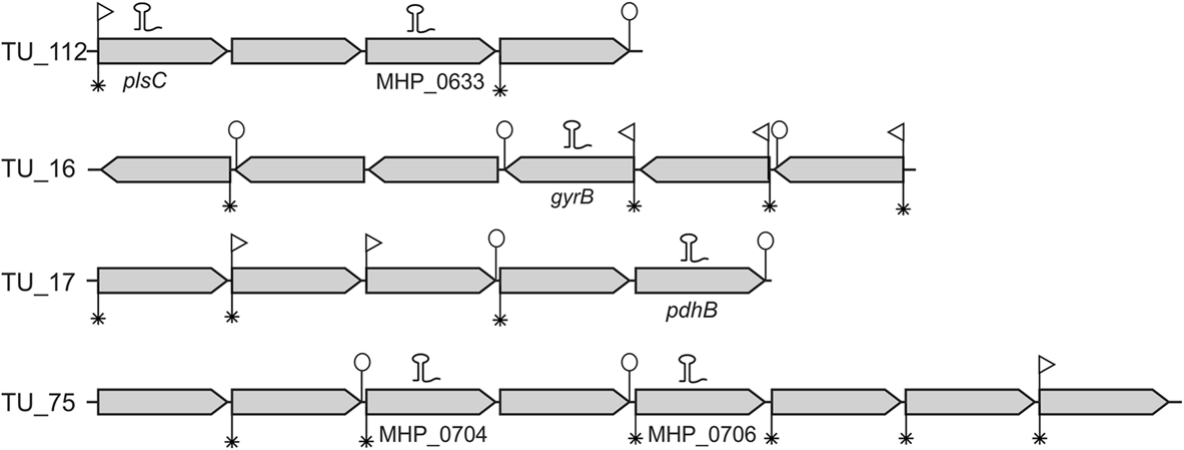
Schematic representation of the presence of the transcription regulatory elements at the TUs with sRNA target genes. TU_112 with the sRNA target genes *plsC* and MHP_0633; TU_16 with the sRNA target gene *gyrB*; TU_17 with the sRNA target gene *pdhB*; TU_75 with the sRNA target genes MHP_0704 and MHP_0706. The hairpin represents the sRNA, the white arrow represents promoter motif, the asterisk represents the DNA repeat sequence and the white circle represents the intrinsic terminator

The presence of promoter motifs, DNA repeat sequences and intrinsic terminators was analyzed both at the sRNA target gene level and also considering their organizational structure as transcriptional units (mCs and TUs). In 16 out of the 145 sRNA target genes (11%), the presence of three regulatory sequences was found: promoter motif, DNA repeat sequence and intrinsic terminator (Fig. 1a). A typical example can be seen in the sRNA target gene *gyrB*, TU_16, as three regulatory sequences were found, suggesting transcriptional regulation independent of the transcription unit (Fig. 2). A detailed analysis of the three regulatory sequences revealed that the DNA repeat was the most prevalent regulatory element in the sRNA target genes, identified in 80 out of 145 (55%) (Fig. 1a). Moreover, this regulatory sequence was present in 20 sRNA target genes as a single putative regulator sequence and in 40 sRNA target genes associated with promoter motifs or intrinsic terminators. However, no regulatory element was found in 29 sRNA target genes (20%), suggesting that the transcription of these genes is associated with the boundaries of the transcription unit (Fig. 1a).

Analysis of the genome organization of the sRNA target genes revealed the presence of at least one regulatory element (promoter motif, DNA repeat sequences or intrinsic terminators) in the majority of the transcription units (Fig. 1b and c). In the seven sRNA target genes organized into mCs, at least one regulatory element was identified (Fig. 1b). Moreover, two mCs, mC_25 (MHP_0357) and mC_30 (MHP_0522), revealed the presence of three transcriptional regulatory elements (Fig. 1b and Additional file 3). The presence of a promoter motif was located in six out of the seven mCs; mC_02 (sRNA target gene MHP_0007) was the only one without promoter motif, yet a DNA repeat sequence and intrinsic terminator was found.

Analysis of TUs containing sRNA target genes also revealed diversity in the presence of regulatory elements. Initially, the analysis considered only the presence of a promoter motif and a repetitive element upstream of the first TU gene and the intrinsic terminator downstream the TU last gene. The presence of a promoter motif was identified in 60 out of the 75 TUs with sRNA target genes, while DNA repeat sequences were identified in 66 TUs and intrinsic terminators in 59 TUs (Fig. 1c). Furthermore, 46 TUs (60%) show the presence of three regulatory elements (Fig. 1c). A typical example can be found in TU_112 (with the *plsC* and MHP_0633 genes, the targets of sRNAs_39 and sRNA_14, respectively) where a promoter motif and repetitive element were found upstream of the TU first gene and an intrinsic terminator downstream of the last gene (Fig. 2; Additional file 3).

Some TUs show an absence of at least one of the transcription regulatory elements upstream of the first TU gene and the intrinsic terminator downstream of the last TU gene. These TUs have alternative transcription regulatory elements in internal genes of the TU. In 10 TUs with sRNA target genes, the promoter motif and DNA repeat were localized upstream of the first gene but no intrinsic terminator was found downstream of the last gene (Fig. 1c). However, in some of these TUs, the presence of internal intrinsic terminators was identified, suggesting that these alternative elements could be related to transcription unit termination. Figure 2 shows TU_16 as an example of a promoter motif and DNA repeat sequence positioned upstream of the first gene of the transcription unit, in addition to the presence of two alternative internal transcription intrinsic terminators.

Another organization profile was found in another eight TUs with sRNA target genes, which represents the presence of a DNA repeat sequence upstream of the first gene and an intrinsic terminator downstream of the last gene of the TU; also, alternative promoters could be localized at internal genes. This profile is illustrated by TU_17 (Fig. 1c; Fig. 2; Additional file 3). Moreover, no regulatory elements (promoter motif, DNA repeat sequences and intrinsic terminators) were found at the 5′ or 3′ of TU_49, TU_75, TU_120 and TU_121 (representing only 5% of all TUs with sRNA target genes). However, internal regulatory sequences were identified in all of these units. Figure 2 shows TU_75 as an example of the presence of alternative regulatory elements in internal genes.

### Differential expression analysis

Aiming to further understand the RNA-based regulation, differential expression analysis by quantitative PCR was utilized to investigate the transcription expression levels of sRNAs and their target genes in three different culture conditions. The number of sRNAs experimentally analyzed took into consideration the difficulties with primers design, as the sequences of the sRNAs located in intergenic regions have a high adenine and thymine content. In total, 19 sRNAs and their corresponding sRNA target genes were subjected to experimental analysis. The presence of amplification was detected for all of the primers utilized and the primer efficiency was greater than 80% for the majority (Additional file 1).

**Fig. 3:**
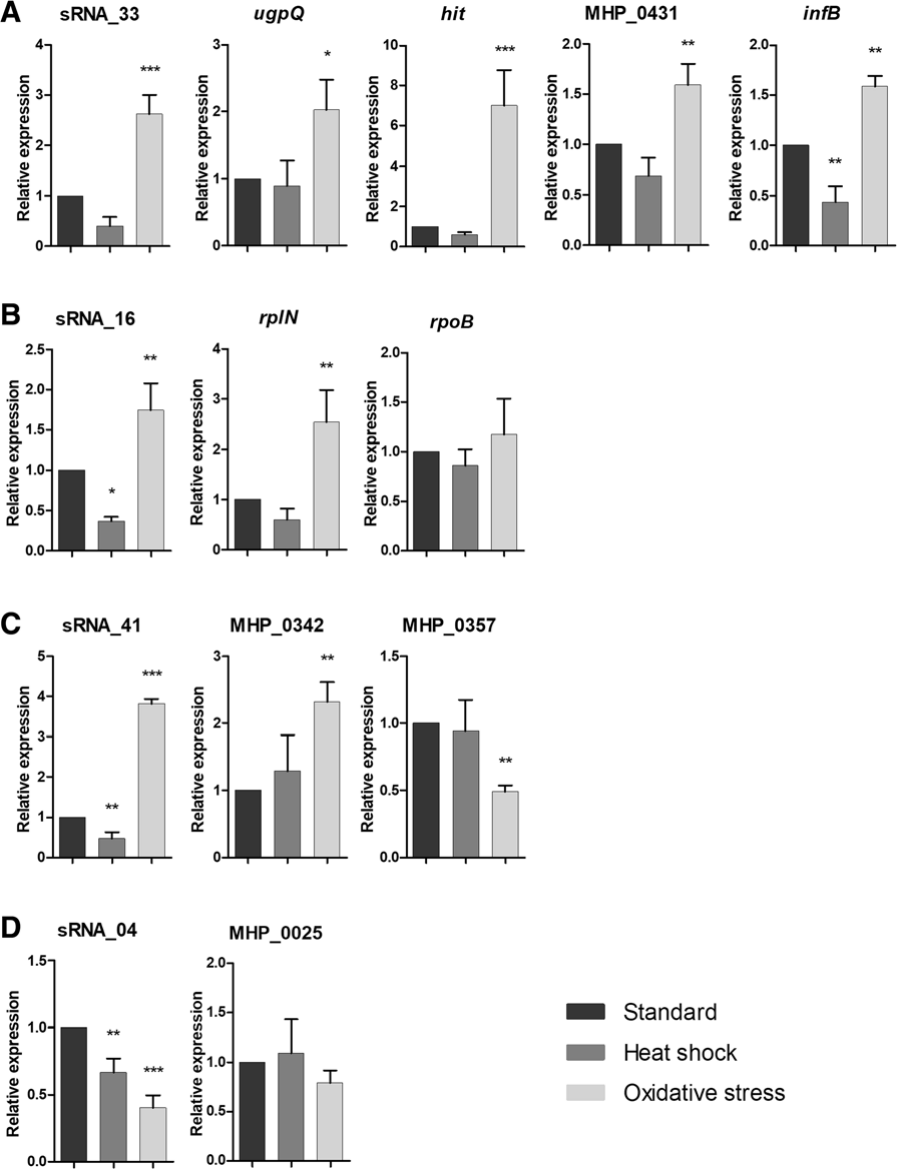
Analysis of relative expression of the sRNAs and their target genes in three culture conditions. **a** sRNA_33 and its target genes *ugpQ*, *hit*, MHP_0431 and *infB*. **b** sRNA_16 and its target genes *rplN* and *rpoB*. **c** sRNA_41 and its target genes MHP_0432 and MHP_0357. **d** sRNA_04 and the target gene MHP_0025. The dark gray represents the standard culture conditions, the medium grey represents the heat shock condition and light grey represents the oxidative stress condition. Data are presented as mean ± standard deviation of three independent experiments. Asterisks indicate statistically significant differences in levels of expression downstream in relation to standard culture condition; *0.01 < *P* < 0.05; **0.001 < *P* < 0.01; ***P < 0.001

Expression analysis of the 19 selected sRNAs revealed that 16 showed differential expression, with seven under heat shock condition and 14 under oxidative stress condition (Table 1). Interestingly, among the 16 sRNAs, five revealed differential expression in both of the conditions tested. The seven sRNAs that are differentially expressed in heat shock condition are down-regulated in relation to the standard condition. Otherwise, of the 14 sRNAs differentially expressed in oxidative stress condition, 12 were up-regulated and two were down-regulated in relation to the standard condition. Only three sRNAs (sRNA_09, sRNA_17 and sRNA_19) showed no differential expression in the analyzed conditions. The relative expression of sRNA_04, sRNA_16, sRNA_33 and sRNA_41 is shown in Fig. 3, while the results of the other sRNAs are in Additional file 3. Table 1 summarizes the results of the differential expression analysis.

**Table 1:** Results of relative expression of sRNAs and their targets in different culture conditions

sRNA	HS^a^	OS^b^	sRNA target gene	HS^a^	OS^b^
sRNA_30	–	up	MHP_0446	–	up
sRNA_33	–	up	*ugpQ*	–	up
			*hit*	–	up
			MHP_0431	–	up
			*infB*	down	up
sRNA_35	–	up	*pdhB*	–	up
sRNA_36	–	up	*deoB*	–	up
sRNA_16	down	up	*rplN*	–	up
			*rpoB*	–	–
sRNA_38	–	up	*gyrB*	down	down
sRNA_39	–	up	MHP_0217	–	–
			*plsC*	–	down
sRNA_14	–	up	*rplC*	down	–
			MHP_0411	down	down
			MHP_0594	–	up
			MHP_0633	down	–
sRNA_40	–	up	*recA*	–	up
			MHP_0646	down	down
sRNA_41	down	up	MHP_0342	–	up
			MHP_0357	–	down
sRNA_4	down	down	MHP_0025	–	–
sRNA_5	down	–	MHP_0476	–	–
sRNA_18	down	up	MHP_0516	–	–
sRNA_34	down	–	MHP_0704	–	–
sRNA_42	down	down	MHP_0498	–	–
sRNA_37	–	up	–	–	–
sRNA_09	–	–	–	–	–
sRNA_17	–	–	–	–	–
sRNA_19	–	–	–	–	–

^a^Differential expression in heat shock condition in relation to the standard condition

^b^Differential expression in oxidative stress condition in relation to the standard condition

Further analysis of the 16 sRNAs that showed differential expression revealed the presence of 36 different predicted sRNA target genes. Aiming to analyze the differential expression of the sRNA target genes, the following criterion was established. The selected sRNA target gene must be the target of a unique differentially expressed sRNA, allowing correlation with the possible action of one of the specific sRNAs in its target genes. Therefore, of the 36 sRNA target genes, 25 sRNA target genes were selected for expression analysis. The sRNA target genes analyzed are presented in Table 1 according to the sRNA interactions. Differential expression was found in 18 sRNA target genes in at least one of the analyzed culture conditions. Six sRNA target genes were down-regulated in the heat shock condition, while, of the 16 sRNA target genes regulated in the oxidative stress condition five were down-regulated and 11 up-regulated.

Usually, the mechanism of regulation by bacterial sRNAs involves the establishment of short, often imperfect base-pair interactions with target mRNAs and could be involved in the regulation of multiple genes. Therefore, the results of the differential expression of the sRNA were correlated with their target genes. Figure 3a shows that sRNA_33 and its target genes (*ugpQ*, *hit*, MHP_431 and *infB*) were all up-regulated in oxidative stress condition. Similar results were found for sRNA_30, sRNA_35 and sRNA_36 and their target genes MHP_446, *pdhB* and *deoB*, respectively (Table 1; Additional file 3). Analyses of the sRNA_16 expression demonstrated a different correlation with the expression of its two target genes (Fig. 3b). The sRNA_16 is down-regulated in heat shock condition and up-regulated in oxidative stress condition. However the sRNA_16 target *rplN* (50S ribosomal protein L14) was up-regulated only under oxidative stress condition while the sRNA_16 target *rpoB* (DNA-directed RNA polymerase beta subunit) showed no differential expression in the tested conditions (Fig. 3b).

Different expression was also found between one sRNA and its target gene, as found for the sRNA_38 and sRNA_39, which showed up-regulation in oxidative stress condition, while their targets were down-regulated under these condition (Table 1; Additional file 3). This suggests that sRNA_38 and sRNA_39 could be involved in suppression of the expression of *gyrB* (DNA gyrase subunit B) and *plsC* (1-acyl-sn-glycerol-3-phosphate acyltransferase) under oxidative stress condition. Another interesting result was found when analyzing the sRNA_14, sRNA_40 and sRNA_41 and its target gene expression (Table 1). The sRNA_41 expression is up-regulated under oxidative stress condition along with its target gene MHP_0342 (hypothetical protein), while the target gene MHP_0357 (amino acid permease) is down-regulated under oxidative stress condition (Fig. 3c). These findings suggest that an sRNA may have different effects on its target genes. Some differentially-expressed sRNAs could not be correlated with the expression levels of their target genes, as found for sRNA_4, sRNA_5, sRNA_18, sRNA_34, and sRNA_42 (Table 1; Fig. 3d; Additional file 3). Although these five sRNAs display differential expression, their target genes showed no significant results compared to the standard conditions tested.

## Discussion

Small bacterial RNAs act as gene expression regulators and are involved in many aspects of bacterial physiology: at the transcriptional, post-transcriptional or translational level [3, 4]. To investigate the putative role of sRNAs in the regulation of gene transcription in *M. hyopneumoniae*, we searched for the presence of transcription regulatory elements in the sRNA target genes and analyzed the expression of sRNAs and their targets under three different culture conditions. We highlight, that the culture medium and conditions used here simulates the conditions at the respiratory tract from pigs, but, as this was an in vitro study, we can only speculate about these findings in in vivo conditions.

The presence of at least one regulatory element (promoter motif, DNA repeat sequence or intrinsic terminator) was identified in 80% of the 145 sRNA target genes. Furthermore, the analysis considering genome organization revealed the presence of regulatory elements in all seven mCs and in 73 of the 77 TUs containing sRNA target genes. Moreover, detailed analysis of the 29 sRNA target genes without regulatory elements revealed the presence of at least one regulatory element positioned at the boundaries of the TU or in other internal genes of the TU. For example, in the gene MHP_0633 from TU_112, no regulatory element was found; however, the presence of a promoter motif and DNA repeat sequences was identified upstream of the first gene of the TU and an intrinsic terminator was identified downstream of the last gene of the TU. In summary, sRNA target genes are preferably distributed in transcription units and all contain regulatory elements in sRNA target genes or the transcriptional unit. Junier et al. [22] showed that the expression of transcription units is dynamically regulated, making it possible to adapt transcription in response to changing conditions. The promoter motifs, DNA repeat sequences and intrinsic terminators have an essential role in the regulation of transcription in bacteria [5]. Siqueira et al. [19] correlated the existence of a different level of transcripts for genes belonging to a transcriptional unit with the presence of internal promoter motifs in the TUs. The presence of transcription regulatory elements in the majority of sRNA target genes suggest that these elements could be related to transcription regulation by base-pairing interactions with sRNA in *M. hyopneumoniae*, similarly to the sRNA regulatory mechanism described in other bacteria species [4, 32, 33].

Previously, Siqueira et al. [23] had demonstrated the transcription of sRNAs in *M. hyopneumoniae* and the differential expression of sRNA_05, which was transcribed in heat shock and oxidative stress conditions, but not under standard conditions, and for sRNA_09, which was only transcribed in standard culture and oxidative stress condition. In contrast, we verified that 16 of the 19 sRNAs tested were differentially expressed and the results obtained for sRNA_05 and sRNA_09 diverge compared with those of Siqueira et al. [23]. The differences in the results are probably related to the methodology used in the present study, which is more accurate. The differential expression in 16 sRNAs supports the notion that the sRNAs contribute to the regulation of gene expression in mycoplasmas. Furthermore, Siqueira et al. [23] identified homologous sequence (100% of sequence identity) of predicted sRNAs in other available *M. hyopneumoniae* strains (J, 7422, 232, 168 and 168-L). Therefore its possible to suggest that these results could be generalized to others *M. hyopneumoniae* strains. In contrast, *M. hyopneumoniae* sRNAs sequences were not found in other Mycoplasma species, suggesting that the sRNAs of mycoplasmas are species-specific. Several studies have established the importance of sRNAs as modulators of gene regulation involved in cell adaptation, responses to changing environments and pathogenesis and other processes [32–34].

Similarly to our work, the use of quantitative PCR to establish the relation between the expression of an sRNA and the effect in the transcriptional regulation of the sRNA target genes was previously described in *Emiliania huxleyi* and *Escherichia coli* [4, 35]. In the present study, we verified the possible role of sRNA in the regulation of gene expression in 10 of the 16 differentially expressed sRNAs; the sRNA target genes were either up- or down-regulated in response to sRNAs under oxidative stress condition in *M. hyopneumoniae*.

The regulation of gene expression by sRNA occurs by several mechanisms in bacteria [32, 34, 36]. Goodson et al. [36] proposed that sRNAs could activate the transcription of their targets by recruiting the bacterial RNA polymerase complex to the promoter region. Interestingly, we have identified promoter motifs in 55 sRNA target genes, which may be used by sRNAs during transcription. Another aspect of sRNA regulation is related to mRNA stability, where the sRNA can increase the stability of the target gene by the protection of the 5’ mRNAs from degradation by exonucleases [32]. However, the down-regulation of gene expression by sRNA could be related to the degradation of mRNA by exonucleases in the response of the base-pairing of sRNAs in the coding sequence region of the target gene [7]. The down-regulation of gene expression was previously demonstrated in *Salmonella* sRNAs MicC and RybB, which increases the degradation of some sRNA target genes through base-pairing within the coding region of mRNAs [37]. However, the mechanisms by which the sRNA act on their target genes still needs to be analyzed and, probably, other factors are involved in the regulation by the sRNAs.

The trans-encoded regulatory RNAs are able to target mRNA via imperfect base-pairing; our results have suggested that different outcomes could be expected from the regulation of the sRNA target genes. Up- or down-regulation was observed when analyzing the expression of sRNA target genes of sRNA_14, sRNA_40 and sRNA_41 (Table 1). The expression of sRNA_41 is up-regulated along with the sRNA target gene MHP_342, while the sRNA target gene MHP_0357 is down-regulated in oxidative stress condition (Fig. 3c). The hybridization site predicted for sRNA_41 with the sRNA target gene MHP_0342 is localized in the regulatory region upstream of the start codon, while the coding region sRNA target gene MHP_0357 base-pairs with sRNA_41. The localization of the sRNA base-pair could explain the different response of sRNA_41 in their sRNA target genes (Additional file 2). The promoter motif identified in the regulatory region of the sRNA target gene MHP_0342 could be related to the up-regulation of transcription found in this gene, while the down-regulation found for the sRNA target gene MHP_0357 could be related to degradation due to base-pairing with sRNA in the coding sequence [7, 36]. These results suggest that the mechanisms of action by sRNAs could be related to the region of hybridization with the target gene, but this needs to be experimentally analyzed.

Other classes of regulatory RNAs have been found in *Mycoplasma*, such as tssRNAs and *cis*-encoded antisense RNAs [24, 25], suggesting that noncoding RNAs identified in *Mycoplasma* possibly control general processes of the cell, as described in other bacteria [38]. Moreover, the sRNAs may act at the translational level by base-pairing in the ribosome binding site (RBS) of their targets [32], or can also be involved in the termination pathway to prevent premature transcription termination, suggesting a broad role of these RNAs [4]. Our analysis in heat shock condition indicates that sRNAs had no effect on the expression of the sRNA target genes and the mechanisms discussed above may be involved in the regulation of sRNA target genes under the tested conditions. Furthermore, other mechanisms of response to heat shock, not related to noncoding RNAs, were identified in mycoplasmas [39, 40].

Regulatory RNAs are extremely diverse. The trans-encoded RNAs, which target genes by imperfect base-pairing, can regulate multiple genes with diverse functions in bacterial physiology [23, 26, 38]. Based on the results of our group, 68% of the total *M. hyopneumoniae* sRNAs can hybridize with more than one mRNA and the target genes are related to different physiological functions. A typical example is the target genes of the sRNA_33 (*ugpQ*, *hit*, MHP_431 and *infB*) that encode proteins involved in different functions; all were up-regulated under oxidative stress condition (Fig. 3).

## Conclusions

In the current study, we speculated the relationship of sRNAs with transcriptional regulation in *M. hyopneumoniae*. The presence of promoter motifs, DNA repeat sequences and intrinsic terminators were observed in the majority of sRNA target genes, suggesting that sRNAs could act by base-pairing with these elements. Moreover, the transcription of both the sRNAs and their target genes was regulated in oxidative stress condition, indicating that sRNAs might be involved in the transcription regulation of their target genes in *Mycoplasma*. Considering the data of sRNA presented in this work, in addition to other classes of previously described noncoding RNAs, we suggest that the small RNAs possibly play a central role in complex global regulatory networks in *Mycoplasma*. However, the mechanism of regulation by sRNA in *M. hyopneumoniae* still needs to be well characterized.

## Additional files

Additional file 1: List of primers used in qPCR. The data presents sequence of primer, the product size and primer efficiency. Amplification efficiency was calculated by LinRegPCR software. (XLSX 15 kb)

Additional file 2: List of the 145 sRNA target genes. The data presents the analysis of the genome organization, presence of promoter motifs, DNA repeat sequences and intrinsic terminators and the region of hybridization with sRNA. (XLSX 50 kb)

Additional file 3: Analysis of relative expression by qPCR of the sRNAs and their target genes in three culture conditions. **A –** The sRNA_14 and their target genes *rplC*, MHP_0411, MHP_0594 and MHP_0633. **B –** The sRNA_39 and their target genes MHP_0217 and *plsC*. **C –** The sRNA_40 and their target genes *recA* and MHP_0646. **D –** The sRNA_30 and their target gene MHP_0446. **E –** The sRNA_35 and their target gene *pdhB*. **F –** The sRNA_36 and their target gene *deoB*. **G –** The sRNA_38 and their target gene *gyrB*. **H –** The sRNA_05 and their target gene MHP_0476. **I –** The sRNA_18 and their target gene MHP_0516. **J –** The sRNA_34 and their target gene MHP_0704. **K -** The sRNA_42 and their target gene MHP_0498. **L –** The sRNA_37. **M –** The sRNA_09. **N –** The sRNA_17. **O –** The sRNA_19. The dark gray represents the standard culture conditions, the medium grey represents the heat shock condition and light grey represents de oxidative stress condition. Data are presented as mean ± standard deviation of three independent experiments. Asterisks indicate statistically significant differences in levels of expression downstream in relation to standard culture condition; *0.01 < *P* < 0.05; **0.001 < *P* < 0.01; ***P < 0.001. (PDF 74 kb)

## Abbreviations

CT: threshold cycle
mC: monocistronic gene
qPCR: quantitative real time PCR
RT: reverse-transcription
sRNA: small RNA
tssRNA: transcription start site-associated RNA
TU: transcriptional unit
