# Supplementary figures and images for: Global analysis of sRNA target genes in Mycoplasma hyopneumoniae
Source: BMC Genomics. 2018 Oct 23;19:767. doi: 10.1186/s12864-018-5136-5 (PMC6199787; doi:10.1186/s12864-018-5136-5)

**A**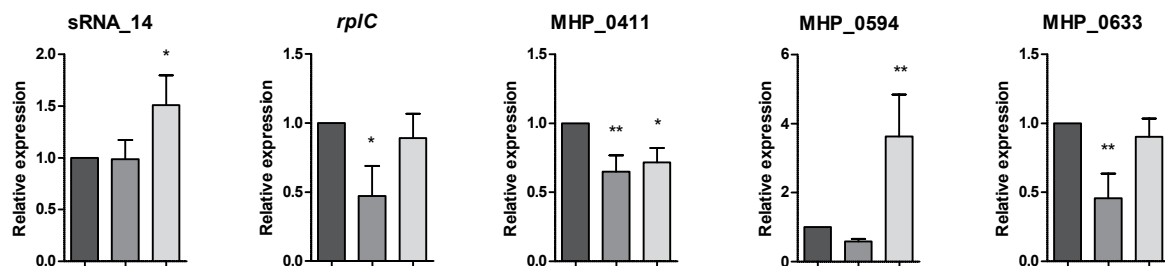**B**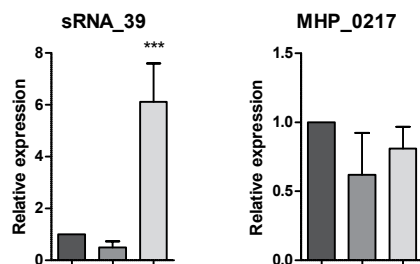**C**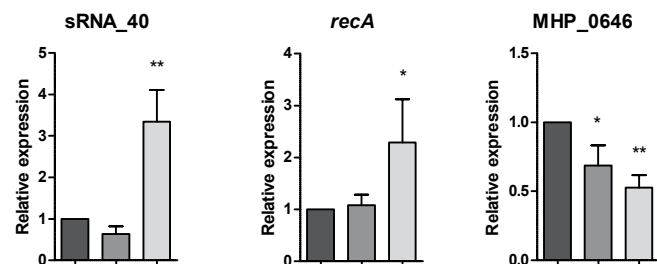**D**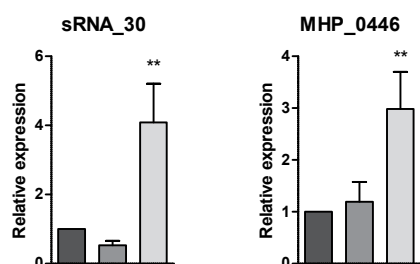**E**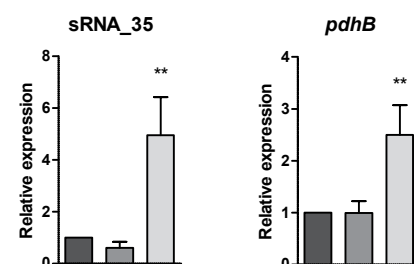**F**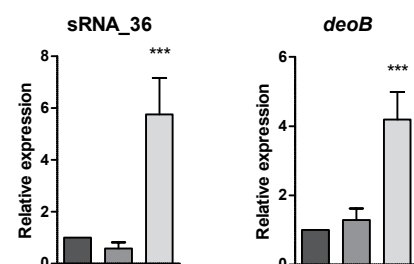**G**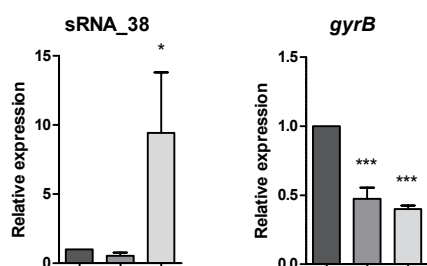**H**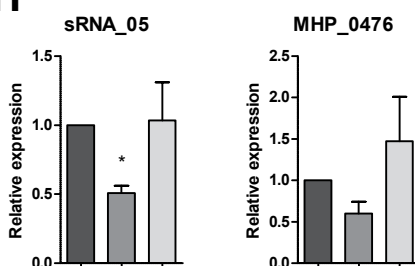**I**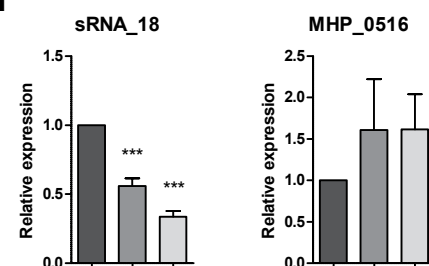**J**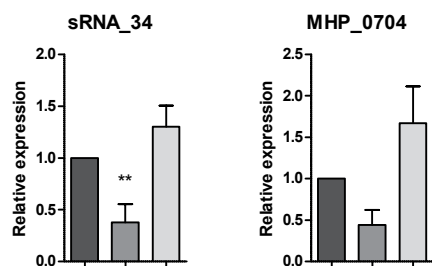**K**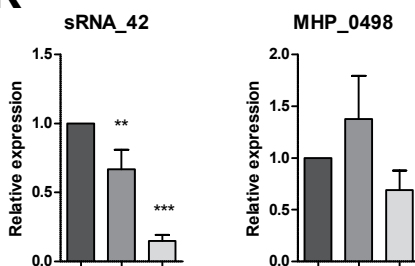**L**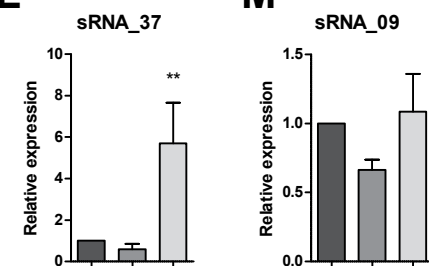**N**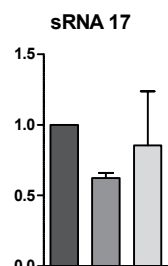**O**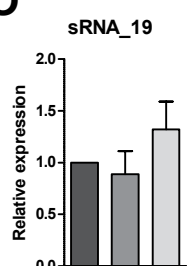

Standard  
Heat Shock  
Oxidative stress

Supplement: Supplementary file 3 — Analysis of relative expression by qPCR of the sRNAs and their target genes in three culture conditions. A – The sRNA_14 and their target genes rplC, MHP_0411, MHP_0594 and MHP_0633. B – The sRNA_39 and their target genes MHP_0217 and plsC. C – The sRNA_40 and their target genes recA and MHP_0646. D – The sRNA_30 and their target gene MHP_0446. E – The sRNA_35 and their target gene pdhB. F – The sRNA_36 and their target gene deoB. G – The sRNA_38 and their target gene gyrB. H – The sRNA_05 and their target gene MHP_0476. I – The sRNA_18 and their target gene MHP_0516. J – The sRNA_34 and their target gene MHP_0704. K - The sRNA_42 and their target gene MHP_0498. L – The sRNA_37. M – The sRNA_09. N – The sRNA_17. O – The sRNA_19. The dark gray represents the standard culture conditions, the medium grey represents the heat shock condition and light grey represents de oxidative stress condition. Data are presented as mean ± standard deviation of three independent experiments. Asterisks indicate statistically significant differences in levels of expression downstream in relation to standard culture condition; *0.01 < P < 0.05; **0.001 < P < 0.01; ***P < 0.001. (PDF 74 kb) [file 12864_2018_5136_MOESM3_ESM.pdf]
